# The value of ultrasound measurement of muscle thickness at different sites and shear wave elastography in Parkinson’s disease with sarcopenia: a pilot study

**DOI:** 10.3389/fnins.2023.1254859

**Published:** 2023-10-13

**Authors:** Minglei Chen, Xiaofang Liu, Qiuwan Liu, Changwei Ding, Ping Zhao, Yingchun Zhang, Chengjie Mao, Chunfeng Liu

**Affiliations:** ^1^Department of Neurology and Clinical Research Center of Neurological Disease, The Second Affiliated Hospital of Soochow University, Suzhou, China; ^2^Department of Neurology, The Third Affiliated Hospital, Sun Yat-sen University, Guangzhou, Guangdong, China; ^3^Department of Neurology, The Second People’s Hospital of Hefei, Hefei Hospital Affiliated to Anhui Medical University, Hefei, Anhui, China; ^4^Department of Ultrasound, The Second Affiliated Hospital of Soochow University, Suzhou, China; ^5^Jiangsu Key Laboratory of Neuropsychiatric Diseases and Institute of Neuroscience, Soochow University, Suzhou, China

**Keywords:** Parkinson’s disease, sarcopenia, muscular ultrasound, muscle thickness, shear wave elastography

## Abstract

**Background:**

Patients with Parkinson’s disease (PD) and sarcopenia often exhibit resilience, frailty, disability, and depression, highlighting the complex and interrelated nature of these conditions.

**Objective:**

Despite the presence of clinical manifestations of muscle atrophy in both PD and sarcopenia, accurately discerning the coexistence of sarcopenia in PD patients remains a challenging task with significant implications for treatment strategies and prognostic assessments. This study aims to elucidate the specific ultrasonic diagnostic parameters associated with PD accompanied by sarcopenia through a comparative analysis of muscle ultrasound parameters in patients with PD, thereby presenting a novel approach for rapid identification of this condition.

**Methods:**

A total of 110 participants were enrolled in this study, including patients with PD and control subjects. Demographic data, clinical characteristics, physical performance tests, appendicular skeletal muscle mass index (ASMI), bioelectrical impedance analysis and muscle ultrasound measurements were collected from all participants. The muscle ultrasound measurements encompassed assessments of muscle thickness, pennation angle and shear wave elastography at various anatomical sites.

**Results:**

Parkinson’s disease patients exhibited decreased muscle strength and physical performance, and increased shear wave elastography value. In PD patients with sarcopenia, body circumference, including calf circumference, mid-arm circumference, Waist-to-Hip Ratio and body mass index (BMI) were all significantly decreased. Biceps brachii muscle thickness (MT) and gastrocnemius MT decreased in PD patients with sarcopenia and low ASMI. Binary logistic regression analysis revealed that male PD patients, BMI and gastrocnemius MT were predictive factors for ASMI in PD patients.

**Conclusion:**

Biceps brachii MT and gastrocnemius MT are important indicators for distinguishing whether PD patients have sarcopenia. Male patients, low BMI and gastrocnemius MT were identified as valid predictors of low ASMI in PD patients. The findings of this study provide important insights into the use of muscle ultrasound in the diagnosis of PD with sarcopenia.

## Introduction

Parkinson’s disease (PD), the second most common neurodegenerative disorder worldwide, is characterized by motor and non-motor symptoms such as motor bradycardia, resting tremor, myotonia, sleep disturbances, constipation, depression, anxiety, and autonomic dysfunction (Vijiaratnam et al., 2021). Sarcopenia, a major contributor to frailty in older adults, is associated with an increased risk of falls, disability, osteoporosis, anxiety, depression, and mortality, critically impacting their quality of life and life expectancy (Yalcin et al., 2017).

Parkinson’s disease and sarcopenia, along with other neurodegenerative diseases such as Alzheimer’s disease, share common pathophysiological pathways, including inflammation, autophagy, oxidative stress, and apoptosis (Vetrano et al., 2018). Moreover, PD and sarcopenia can interact with each other, forming a vicious cycle wherein sarcopenia accelerates the age-related process of muscle atrophy in PD patients, eventually promoting the neurodegenerative processes underlying PD (Lee et al., 2016). Additionally, PD patients with sarcopenia are at an increased risk of falls, cognitive disorders, and depression (Lima et al., 2020). Early screening and diagnosis of sarcopenia, followed by appropriate intervention, are essential for PD patients to manage their condition effectively (Wilson et al., 2019).

Muscle strength and physical performance can be evaluated using several methods such as handgrip strength, 6-meter gait speed (GS), short physical performance battery (SPPB), and the five times sit-to-stand test (FTSTS) (Chen et al., 2020). However, the determination of skeletal muscle mass primarily relies on imaging techniques such as computed tomography (CT), magnetic resonance imaging (MRI), dual-energy X-ray absorptiometry (DXA), bioelectrical impedance analysis (BIA), and ultrasound examinations (Lustgarten and Fielding, 2011; Cruz-Jentoft et al., 2019). Among these, ultrasound assessment for sarcopenia stands out due to its advantageous features, such as high portability, low cost, no ionizing radiation, high reproducibility, and broad clinical application (Perkisas et al., 2021). Compared with the high cost of CT and MRI, the radiation exposure of CT and DXA, and the overestimation of muscle mass by BIA, muscle ultrasound shows many advantages, such as intuitive visualization, simple operation, low cost, real-time assessment, portability, and no radiation. Since the 1990s, some scholars have begun to use ultrasound to quantitatively evaluate the functional state of muscles and apply the analysis results to biological force (Zheng et al., 2006). Guo et al. (2008) used the one-dimensional myoacoustic signal (A-scan ultrasound) to study the state changes of forearm muscles during contraction, and proved that it was a reliable signal for analyzing muscle activity. Two-dimensional myoacoustic signals (B-mode ultrasound) can provide richer and more intentional myoacoustic signals than one-dimensional myoacoustic signals. Information on muscle fiber length, muscle thickness (MT) and pennation angle are useful for further analysis and assessment of muscle function. Shear wave elastography (SWE), a novel technique for quantitative assessment of tissue stiffness, shows great potential in detecting muscle stiffness (Ewertsen et al., 2016; Ding et al., 2021). Nevertheless, there is limited research on the application of muscle ultrasound in PD patients. Thus, this study aims to investigate the potential of brachioradialis SWE, biceps brachii SWE, biceps brachii MT, gastrocnemius fascicle length, gastrocnemius MT, gastrocnemius pennation angle, gastrocnemius resting SWE, and gastrocnemius dorsiflexion SWE in PD patients.

The physiological characteristics vary in different genders. The muscle mass of males is greater than that of females, which is reflected by the MT in muscle morphology. Nishihara et al. (2018) used ultrasound examination to measure the thickness of the rectus femoris and vastus intermedius of the elderly in the community and established a normal reference range, and found that the MT of men was greater than that of women. Another study used ultrasound to measure the MT of 65 healthy people and found that among many influencing factors, gender had the most significant effect on MT (Abraham et al., 2020). We collected data from both PD patients and control subjects and analyzed the correlation between these ultrasonic parameters and sarcopenia to provide a theoretical basis for future ultrasound applications in PD patients with sarcopenia. Differences in ultrasonic parameters between different genders were also assessed in PD patients with sarcopenia.

## Materials and methods

### Subjects

The study sample consisted of 68 PD patients (age range: 51–79 years) who received inpatient and outpatient care at the Second Affiliated Hospital of Soochow University between December 2020 and July 2022. The diagnosis of PD was established based on the Movement Disorders Society and United Kingdom PD Brain Bank criteria, and patients who failed to meet the diagnostic criteria for idiopathic PD were excluded from the study. The Research Ethics Committee of the hospital approved the study, and all participants provided written informed consent (registration number: LK-2018-061-01). Additionally, 42 control subjects (age range: 50–78 years) from the community were recruited as the control group, matching the PD group’s age and gender. The control group inclusion criteria mandated the absence of any history of diseases affecting gait performance such as PD, cerebrovascular disease, depression, dementia, vestibular diseases, or orthopedic diseases and the capability to comply with medical instructions. All patients underwent interviews and evaluations by the study investigators.

### Clinical data collection

**FIGURE 1:**
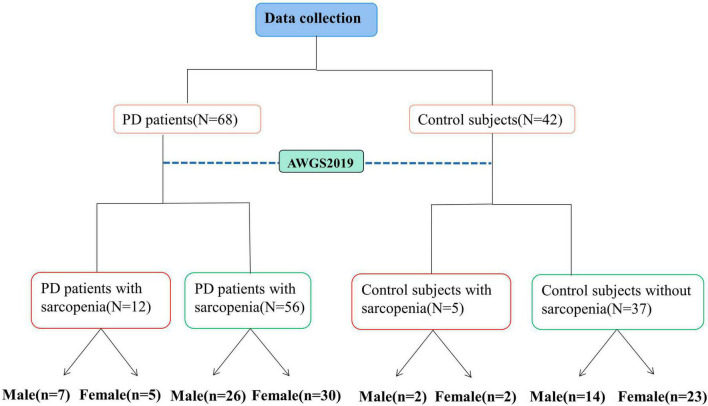
Flow chart of multiple groups. PD, Parkinson’s disease.

The demographic characteristics of all participants were collected along with measurements of height, weight, mid-arm circumference (MAC), calf circumference (CC), waist circumference (WC), and hip circumference on an empty stomach in the morning. Body mass index (BMI) and Waist-to-Hip Ratio (WHR) were calculated using these measurements, where BMI = weight (kg)/height^2^ (m^2^) and WHR = waist circumference/hip circumference. Nutritional status was assessed using the Mini-Nutritional Assessment (MNA). PD patients’ mental behavior, daily living ability, and motor symptoms were evaluated using the Movement Disorders Society’s revised Unified PD Rating Scale (MDS-UPDRS I, II, III) (Poewe et al., 2017). The severity of the disease was assessed with the Hoehn–Yahr (H-Y) stage, and the quality of daily life was assessed with the Activities of Daily Living Scale (ADL) and the 8-item Parkinson’s Disease Questionnaire (PDQ-8). Cognitive function was evaluated using the Montreal Cognitive Assessment (MoCA) and Mini-Mental State Examination (MMSE), while depressive and anxiety symptoms were assessed using the Hamilton Depression Rating Scale-24 (HAMD-24) and Hamilton Anxiety Scale (HAMA), respectively. Moreover, constipation score was used to assess the severity of symptoms. All evaluations were performed by the same physician to minimize inter-individual variability, and all PD patients were evaluated in the OFF state. The flowchart depicted in Figure 1 illustrates the process of data collection and grouping for this study.

### Assessment of muscle strength

Handgrip strength of the dominant hand was measured 3 times using a dynamometer (WCS-100, Nantong, China), with an interval of 1 min between each measurement. The mean value was recorded.

### Assessment of skeletal muscle mass

BIA was performed to estimate skeletal muscle mass (Inbody 720, Biospace Ltd., Seoul, Korea).

### Assessment of physical performance

All subjects were assessed by a trained physician for GS, FTSTS, SPPB (including balance test, 4 meter walking speed, and chair standing). All assessments were conducted in a quiet room dedicated to clinical assessment (Mijnarends et al., 2013).

### Assessment of sarcopenia

The cut-off values recommended by the Asian Sarcopenia Working Group (AWGS) 2019 (Chen et al., 2020) were used as diagnostic criteria. The SARC-calf was used to evaluate sarcopenia risk. It contains six objects: 1, strength; 2, assistance in walking; 3, rising from a chair; 4, climbing stairs; 5, falls; and 6, calf circumference (CC). The first five objects were scored from 0 to 2. Men and women had a CC threshold of 34 and 33 cm, respectively. If the measurement exceeded the critical value, CC was scored as 0. If it fell below the cutoff, a score of 10 was assigned. A score ≥ 11 indicated sarcopenia. Appendicular skeletal muscle mass index (ASMI): male < 7.0 kg/m^2^, female < 5.7 kg/m^2^ was low muscle mass. Men with handgrip strength <26 kg and women with handgrip strength < 18 kg belonged to the low handgrip strength group. If low muscle mass was present in conjunction with low handgrip strength or GS (<1.0 m/s) or FTSTS (≥12 s) or SPPB (≤9) the subject belonged to the sarcopenia group. Using these guidelines, PD patients were divided into PD with sarcopenia and PD without sarcopenia, while the control subjects were divided into controls with sarcopenia and controls without sarcopenia.

### Ultrasonographic evaluation

All ultrasound data were acquired by an expert ultrasound diagnostician using a Supersonic Aixplore ultrasound diagnostic instrument equipped with SWE imaging technology (Sonco, France) and an L4-15 linear array ultrasound probe. During the examination, the physician held the probe suspended perpendicular to the bed and in light contact with the skin to minimize pressure on the muscles.

Biceps brachii MT was measured bilaterally with the participants lying supine and arms naturally positioned on either side of the body with palms up. The long axis of the probe was placed in the middle and upper third of the muscle starting and ending points, perpendicular to the long axis of the upper arm. Images of the medial head of the gastrocnemius were obtained from participants lying prone with their legs extended and feet hanging off the edge of the examination table at the most bulky area of the gastrocnemius. Gastrocnemius MT was measured as the distance between the upper and deeper aponeurosis, pennation angle was measured as the angle of insertion of muscle fascicles into the deep aponeurosis, and fascicle length was measured as the length of the fascicular path between the superficial and deep aponeurosis (Kuyumcu et al., 2016). All data measurements were conducted bilaterally by the same expert ultrasound diagnostician for consistency.

The probe was switched to the SWE mode, and the sampling frame was set as 10 mm x 10 mm in the area of interest with a depth of 1.2 cm. After the image was stabilized, the Q-box was started for shear wave elasticity measurement. When the diameter of the elastic parameter measurement circle was 3 mm, the mean value of Young’s modulus of the muscle was obtained (Ewertsen et al., 2016). Brachioradialis SWE, biceps brachii SWE, gastrocnemius resting SWE (KPa), and gastrocnemius dorsiflexion SWE (KPa) were recorded, respectively. All subjects were instructed to remain as relaxed as possible throughout all testing (approximately 30–60 s) as shown in Supplementary Figure 1.

### Statistical analysis

SPSS 26.0 (IBM, USA) statistical software was used for data processing and analysis. Measurement data conforming to normal distribution were calculated as mean ± standard deviation (x ± s), and measurement data with non-normal distribution were expressed as median (quartile) [M (P25, P75)]. Student’s *t* test or Mann-Whitney test were used to compare MT and pennation angle between patients with and without sarcopenia. Spearman correlation analysis was used to evaluate the correlation between age and other parameters, and to evaluate the correlation between MT, elasticity value, feathery angle and muscle mass, handgrip strength, walking speed and SPPB. Age, sex, BMI, MT, elasticity, and pennation angle were included in binary logistic regression analysis to analyze the predictive factors of muscle mass loss. *P* < 0.05 was considered statistically significant.

## Results

### Comparison of ultrasound data between different sides of limbs within different groups

Statistical analysis was performed to investigate differences in ultrasonic measurement data between the side of onset and the other side of limbs in the PD group. Additionally, we analyzed the differences in ultrasound data between the dominant and non-dominant limbs in the control group. There were no statistically significant differences in bilateral muscular ultrasound measurements between the PD and control groups, except for brachioradialis SWE in the control group and biceps brachii SWE in the PD group (*P* < 0.05), as shown in Supplementary Tables 1, 2. Therefore, muscular ultrasound measurement data on the side of onset in the PD group and the dominant limbs in the control group were analyzed.

### Clinical characteristics between the PD and control groups

The age, sex ratio and BMI were not significantly different between the PD and control groups. Furthermore, ADL, handgrip strength, GS, FTSTS, and SPPB were significantly impaired in the PD group compared to the control group (*P* < 0.05), indicating an overall reduction in physical function, and muscle strength in PD patients. Moreover, brachioradialis SWE and biceps brachii SWE were elevated, and gastrocnemius dorsiflexion SWE was decreased in the PD group compared to the control group (*P* < 0.05). These results are presented in Table 1. Similarly, as shown in Supplementary Tables 3, 4, both female and male PD patients without sarcopenia exhibited higher brachioradialis SWE, biceps brachii SWE, GS, FTSTS, or SPPB impairments than female and male control subjects, respectively. Additionally, female and male PD patients had higher constipation scores.

**TABLE 1:** Clinical characteristics of participants.

Clinical demographics	PD (*n* = 68)	Control subjects (*n* = 42)	*p*
Age (year)^b^	65.25 ± 7.26	62.81 ± 6.74	0.081
Male, *N* (%)^a^	33 (48.5)	17 (40.5)	0.679
Height (cm)^b^	161.96 ± 7.08	159.04 ± 8.16	0.05
Weight (kg)^c^	61.95 (56.23, 69.65)	61.45 (52.05, 71.35)	0.784
BMI^b^	23.87 ± 3.16	24.65 ± 3.21	0.213
WHR^b^	0.87 ± 0.04	0.88 ± 0.04	0.116
MAC(cm)^b^	27.92 ± 2.40	28.07 ± 2.61	0.755
WC(cm)^b^	86.44 ± 10.12	84.33 ± 8.29	0.259
CC(cm)^b^	34.77 ± 2.93	34.92 ± 3.27	0.810
MNA^c^	12 (11, 13)	13 (12, 13)	0.216
ADL^c^	14 (14, 17)	14 (14, 14)	**0.000**
Hypertension, *N* (%)^a^	13 (11.8)	9 (16.4)	0.612
Type 2DM, *N* (%)^a^	1 (0.9)	9 (8.2)	0.114
Alcohol, *N* (%)^a^	9 (8.2)	13 (11.8)	0.768
Smoking, *N* (%)^a^	9 (8.2)	5 (4.5)	0.839
Tea/coffee, *N* (%)^a^	10 (9.1)	11 (10.0)	0.322
ASMI^b^	6.89 ± 0.94	6.87 ± 0.93	0.951
Handgrip strength (kg)^b^	23.32 ± 7.39	27.78 ± 7.39	**0.003**
SPPB^c^	10.05 (9, 12)	12 (11, 12)	**0.000**
GS^c^	0.90 (0.77, 1.02)	1.01 (0.92, 1.15)	**0.000**
FTSTS^c^	11.44 (9.67, 14.43)	8.97 (7.69, 11.30)	**0.000**
Brachioradialis SWE (KPa)^b^	37.09 ± 9.17	30.08 ± 6.05	**0.000**
Biceps brachii SWE (KPa)^b^	67.60 ± 32.69	40.65 ± 11.99	**0.000**
Biceps brachii MT(cm)^b^	2.67 ± 0.45	2.67 ± 0.50	0.990
Gastrocnemius fascicle length (cm)^b^	4.32 ± 0.65	4.46 ± 0.50	0.213
Gastrocnemius MT(cm)^b^	1.48 ± 0.21	1.56 ± 0.27	0.088
Gastrocnemius pennation angle (°)^b^	22.86 ± 4.29	21.29 ± 3.59	0.490
Gastrocnemius resting SWE (KPa)^b^	17.88 ± 22.38	17.10 ± 13.53	0.840
Gastrocnemius dorsiflexion SWE (KPa)^b^	161.18 ± 69.48	220.02 ± 46.27	**0.000**

Values are expressed as *n* (%), mean ± standard deviation or median (interquartile range). ^a^Pearson’s chi-square test. ^b^Student’s *t*-test. ^c^Mann–Whitney U test. *P* < 0.05 were considered statistically significant. Bold values highlight the significant difference.

BMI, body mass index; WHR, Waist-to-Hip Ratio; MAC, mid-arm circumference; WC, waist circumference; CC, calf circumference; MNA, Mini-Nutritional Assessment; ADL, Activity of Daily Living Scale; Type 2DM, type-2 diabetes mellitus; ASMI, appendicular skeletal muscle index; SPPB, short physical performance battery; GS, 6-metre gait speed; FTSTS, five times sit-to-stand test; MT, muscle thickness; SWE, shear wave elastography.

### Comparison of clinical characteristics and muscle ultrasonic parameters between PD patients with and without sarcopenia

Based on the AWGS2019 criteria, 12 of 68 PD patients (17.6%) were classified into the PD with sarcopenia group. Compared with PD patients without sarcopenia, as shown in Supplementary Table 5 PD patients with sarcopenia exhibited a lower CC, WC, MAC, BMI, weight, MNA score, ASMI, WHR, GS and gastrocnemius MT, accompanied by an increase in SARC-calf and UPDRS I. Due to the gender-related differences in sarcopenia and muscle ultrasound data, a gender-matched group was selected for subsequent analysis. As shown in Table 2, the female patients with PD and sarcopenia demonstrated a decrease in CC, MAC, BMI, ASMI, weight, WHR, MNA score, and gastrocnemius MT, while SARC-calf increased. Similarly, Table 3 shows a decrease in CC, WC, MAC, BMI, ASMI, weight, WHR, GS, MNA score, and biceps brachii MT, accompanied by an increase in SARC-calf and UPDRS I score, among male patients with PD and sarcopenia.

**TABLE 2:** Clinical characteristics of female PD with sarcopenia and without sarcopenia.

Clinical demographics	PD-s (*n* = 5)	PD-Ns (*n* = 30)	*p*
Age (year)^b^	67.60 ± 10.21	62.93 ± 6.90	0.200
SARC-Calf^b^	10.60 ± 5.73	3.13 ± 4.61	**0.003**
CC(cm)^b^	30.60 ± 1.52	34.03 ± 2.61	**0.008**
WC(cm)^b^	78.50 ± 10.23	85.85 ± 10.26	0.148
MAC(cm)^b^	25.40 ± 1.67	27.65 ± 2.29	**0.044**
Height (cm)^b^	158.20 ± 4.71	157.04 ± 5.36	0.654
Weight (kg)^b^	48.74 ± 2.21	60.42 ± 7.17	**0.000**
WHR^b^	0.83 ± 0.04	0.88 ± 0.04	**0.010**
BMI^b^	19.78 ± 1.55	24.40 ± 2.40	**0.000**
UPDRS I^b^	4.00 ± 1.87	3.23 ± 1.93	**0.414**
PDQ-8^b^	5.40 ± 4.62	3.63 ± 4.13	0.389
ADL^b^	20.80 ± 9.55	16.10 ± 4.01	0.336
MNA^c^	9.00 (8.50, 11.00)	12.50 (12.00, 13.00)	**0.000**
Constipation score^b^	7.00 ± 5.92	6.6 ± 4.51	0.861
Handgrip strength^b^	15.65 ± 7.09	19.89 ± 5.00	0.108
FTSTS^c^	21.27 ± 11.83	11.76 ± 3.12	0.147
GS^c^	0.72 (0.64, 0.85)	0.90 (0.84, 1.03)	**0.187**
SPPB^c^	9.00 (5.00, 10.5)	11.00 (9.00, 12.00)	0.127
ASMI^b^	5.30 ± 0.37	6.45 ± 0.53	**0.000**
Brachioradialis SWE (KPa)^b^	40.46 ± 12.44	35.21 ± 9.70	0.288
Biceps brachii SWE (KPa)^b^	67.44 ± 45.93	65.39 ± 30.73	0.898
Biceps brachii MT (cm)^b^	2.24 ± 0.48	2.49 ± 0.42	0.236
Gastrocnemius fascicle length (cm)^b^	4.00 ± 0.32	4.32 ± 0.73	0.089
Gastrocnemius MT (cm)^b^	1.20 ± 0.12	1.47 ± 0.16	**0.000**
Gastrocnemius pennation angle (°)^b^	20.77 ± 3.77	23.21 ± 4.72	0.282
Gastrocnemius resting SWE (KPa)^b^	16.64 ± 14.28	18.43 ± 23.13	0.868
Gastrocnemius dorsiflexion SWE (KPa)^b^	191.86 ± 61.93	150.19 ± 66.75	0.201

Values are expressed as mean ± standard deviation or median (interquartile range). ^b^Student’s *t*-test. ^c^Mann–Whitney U test. *P* < 0.05 were considered statistically significant. Bold values highlight the significant difference.

PD-s, Parkinson’s Disease with sarcopenia; PD-Ns, Parkinson’s Disease without sarcopenia; CC, calf circumference; WC, waist circumference; MAC, mid-arm circumference; WHR, Waist-to-Hip Ratio; BMI, body mass index; UPDRS I, Unified Parkinson’s Disease Rating Scale part I; PDQ-8; the 8-item Parkinson’s Disease Questionnaire; ADL, Activity of Daily Living Scale; FTSTS, five times sit-to-stand test; GS, 6-metre gait speed; ASMI, appendicular skeletal muscle index; SPPB, short physical performance battery; MNA, Mini-Nutritional Assessment; MT, muscle thickness; SWE, shear wave elastography.

**TABLE 3:** Clinical characteristics of male PD with sarcopenia and without sarcopenia.

Clinical demographics	PD-s (*n* = 7)	PD-Ns (*n* = 26)	*p*
Age (year)^b^	69.29 ± 7.30	66.38 ± 6.56	0.317
SARC-Calf^b^	7.71 ± 5.31	1.08 ± 2.21	**0.016**
CC(cm)^b^	32.79 ± 1.47	36.96 ± 2.01	**0.000**
WC(cm)^b^	78.79 ± 9.42	90.71 ± 8.11	**0.002**
MAC(cm)^b^	25.93 ± 1.17	29.25 ± 2.07	**0.000**
Height (cm)^b^	166.34 ± 2.75	167.19 ± 5.48	0.699
Weight (kg)^b^	56.61 ± 4.65	70.05 ± 7.35	**0.000**
WHR^b^	0.83 ± 0.03	0.88 ± 0.04	**0.002**
BMI^b^	20.44 ± 1.40	24.97 ± 3.31	**0.000**
UPDRS I^b^	5.00 ± 2.89	2.85 ± 1.93	**0.025**
PDQ-8^b^	4.29 ± 3.90	3.62 ± 4.18	0.705
ADL^b^	16.43 ± 3.65	15.62 ± 3.41	0.584
MNA^c^	11.00 (10.00, 12.00)	13.00 (11.75, 13.00)	**0.031**
Constipation score^b^	4.71 ± 3.40	6.27 ± 4.84	0.433
Handgrip strength^b^	24.92 ± 6.30	28.33 ± 6.87	0.246
FTSTS^c^	15.80 ± 9.61	11.92 ± 5.60	0.175
GS^c^	0.77 (0.64, 0.85)	0.94 (0.84, 1.03)	**0.014**
SPPB^c^	9.00 (8.00, 11.00)	10.5 (8.00, 12.00)	0.451
ASMI^b^	6.67 ± 0.21	7.75 ± 0.68	**0.000**
Brachioradialis SWE (KPa)^b^	37.01 ± 8.53	38.62 ± 8.07	0.647
Biceps brachii SWE (KPa)^b^	66.21 ± 25.58	70.56 ± 35.48	0.765
Biceps brachii MT(cm)^b^	2.62 ± 0.26	2.99 ± 0.32	**0.009**
Gastrocnemius fascicle length (cm)^b^	4.11 ± 0.72	4.45 ± 0.56	0.194
Gastrocnemius MT (cm)^b^	1.40 ± 0.13	1.57 ± 0.22	0.068
Gastrocnemius pennation angle (°)^b^	22.31 ± 5.65	23.01 ± 3.51	0.685
Gastrocnemius Resting SWE (KPa)^b^	12.86 ± 4.39	18.82 ± 25.99	0.554
Gastrocnemius dorsiflexion SWE (KPa)^b^	166.60 ± 68.67	166.50 ± 75.20	0.997

Values are expressed as mean ± standard deviation or median (interquartile range). ^b^Student’s *t*-test. ^c^Mann–Whitney U test. *p* < 0.05 were considered statistically significant. Bold values highlight the significant difference.

PD-s, Parkinson’s Disease with sarcopenia; PD-Ns, Parkinson’s Disease without sarcopenia; CC, calf circumference; WC, waist circumference; MAC, mid-arm circumference; WHR, Waist-to-Hip Ratio; BMI, body mass index; UPDRS I, Unified Parkinson’s Disease Rating Scale part I; PDQ-8; the 8-item Parkinson’s Disease Questionnaire; ADL, Activity of Daily Living Scale; FTSTS, five times sit-to-stand test; GS, 6-metre gait speed; ASMI, appendicular skeletal muscle index; SPPB, short physical performance battery; MNA, Mini-Nutritional Assessment; MT, muscle thickness; SWE, shear wave elastography.

### Comparison of muscle ultrasonic parameters in PD patients with and without low ASMI

**FIGURE 2:**
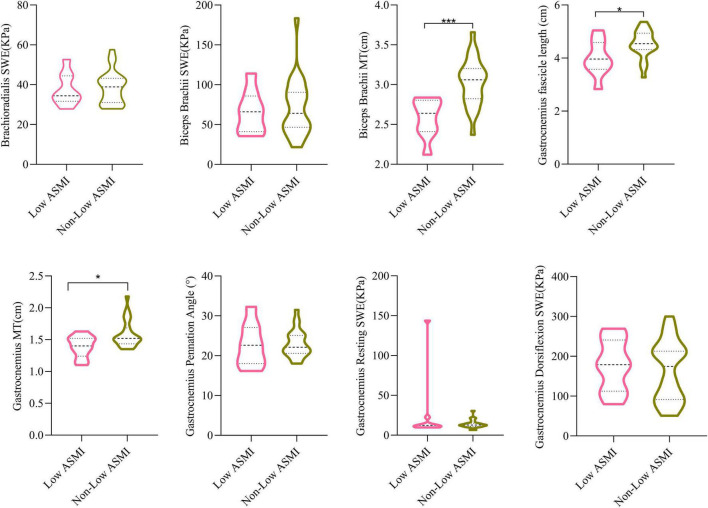
The comparisons of muscle ultrasound parameters of low ASMI PD and non-low ASMI male PD patients. Student’s *t*-test. MT, muscle thickness; SWE, shear wave elastography; ASMI, appendicular skeletal muscle index. *P < 0.05, ***P < 0.001.

To better elucidate the significance of ultrasound data in assessing muscle mass, we categorized male PD patients into two groups based on ASMI levels: a low ASMI group consisting of 9 individuals and a normal ASMI group consisting of 24 individuals. As the number and composition of female PD patients with and without low ASMI remained unchanged from those with and without sarcopenia, we did not proceed with further statistical analysis. We subsequently reanalyzed the ultrasound data for both groups. As shown in Figure 2, biceps brachii MT, gastrocnemius MT and gastrocnemius fascicle length were smaller in the low-ASMI group (*P* < 0.05).

### Correlation analysis of muscle ultrasonic parameters in female PD patients and male PD patients

**FIGURE 3:**
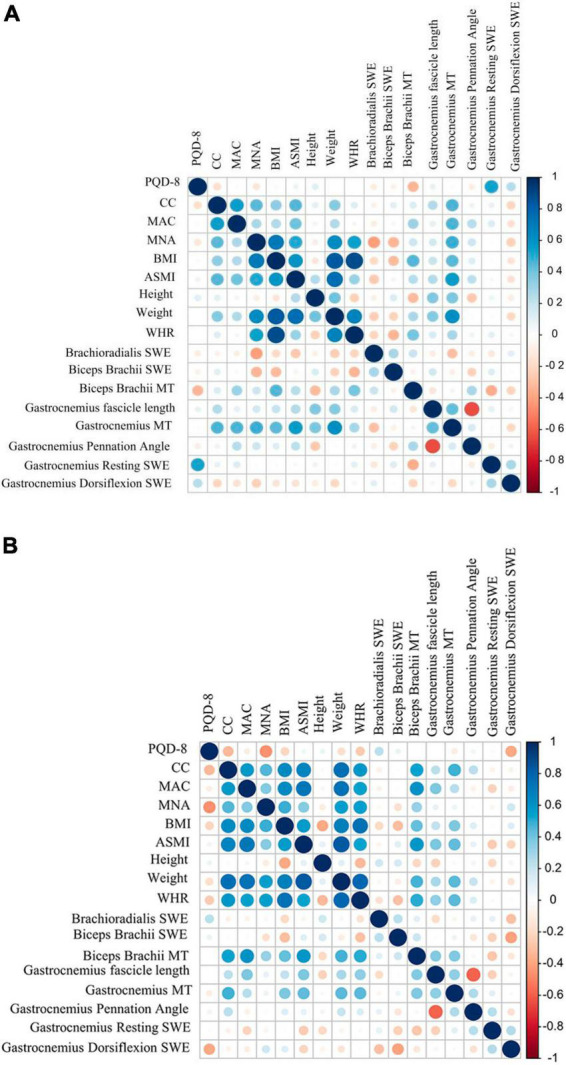
Correlation analysis results between US parameters with different Clinical characteristics in PD patients. **(A)** female PD. **(B)** Male PD. MAC, mid-arm circumference; MT, muscle thickness; PDQ-8, the 8-item Parkinson’s Disease Questionnaire; CC, calf circumference; MAC, mid-arm circumference; BMI, body mass index; ASMI, appendicular skeletal muscle index; WHR, Waist-to-Hip Ratio; MNA, Mini-Nutritional Assessment.

In female PD patients, gastrocnemius MT was significantly positively correlated with CC, MAC, MNA score, BMI, ASMI, and weight (Figure 3A). As shown in Figure 3B, biceps brachii MT was significantly positively correlated with ASMI, CC, MAC, MNA score, BMI, weight, and WHR in male PD patients. Similarly, gastrocnemius MT was also positively correlated with ASMI, weight, and WHR in male PD patients.

### Binary logistic regression analysis of PD patients with low ASMI

**FIGURE 4:**
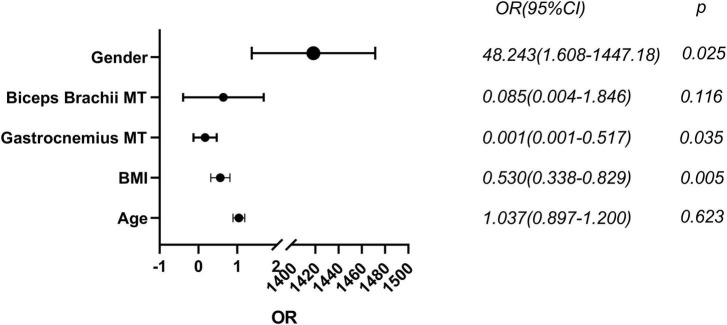
Binary logistic regression analysis of low muscle mass in male PD. MT, muscle thickness; BMI, body mass index. Statistically significant (*p* < 0.05).

In all 68 PD subjects, with age, gender, BMI, and muscle ultrasonic parameters included, gender (OR (95% CI): 48.243 (1.608–1447.18), *P* < 0.05), BMI (OR (95% CI): 0.530 (0.338–0.829), *P* < 0.01) and gastrocnemius MT (OR (95% CI): 0.001 (0.001–0.517), *P* < 0.05) were found to be effective predictors of low ASMI (Figure 4).

## Discussion

As mentioned previously, ultrasound is a good method for assessing muscle mass (Abe et al., 2016). However, there are few previous studies on muscular ultrasound and SWE in the assessment of PD with sarcopenia. Here we measured MT, fascicle length, pennation angle and SWE values of PD patients bilaterally by ultrasound and compared them with the muscle mass assessed by BIA to investigate the value of ultrasound measurement in PD with sarcopenia.

Ultrasound measurement data are likely to differ bilaterally, and some studies have reported that MT as well as cross-sectional area is usually greater in the dominant limb (Barotsis et al., 2020). Hence, we first performed a comparison of bilateral ultrasound data in PD patients and control subjects, and the results showed that there were no differences in various ultrasound indices between the different side-of-onset in PD patients. Similarly, in this study, there was no difference in the muscular ultrasound indices between the dominant side and non-dominant side in the control group. In our study, the prevalence of sarcopenia in patients with PD was 17.6%. A previous systematic review and meta-analysis of population-based studies estimated the overall prevalence of sarcopenia in healthy adults under 60 years of age to be 10% (Shafiee et al., 2017), and a recent systematic review also showed that sarcopenia was more likely to occur in patients with PD compared to controls, and older PD patients are even more likely to develop sarcopenia than older controls (Ponsoni et al., 2023). Possible reasons for the high prevalence of sarcopenia in patients with PD include the reduction in the number of motor neurons, which is a common feature of sarcopenia and PD (Krenovsky et al., 2020). However, PD patients may experience nausea, dyspepsia, constipation, medication side effects, dysphagia, anorexia and depression leading to reduced energy intake, and weight loss is very common in the later stages of PD (Grayson, 2016).

This study showed that PD patients with lower weight, BMI, and WHR may be more likely to develop sarcopenia. Further confirmation is required to ascertain whether patients with sarcopenia experience weight loss, changes in WHR, and alterations in BMI during the progressive exacerbation of sarcopenia. Furthermore, ADL, handgrip strength, 6-meter gait speed, FTSTS, and SPPB were worse in the PD group than in the control group, which is consistent with our perception that PD patients have reduced motor ability and decreased physical performance. A recent systematic analysis found that both elderly men and women with decreased BMI had a significantly increased risk of developing low ASMI (Graf et al., 2017), and underweight patients had a severe decrease in muscle mass. Our study also revealed that a higher BMI can prevent the occurrence of low muscle mass. As shown in Supplementary Table 6, we observed a significant reduction in gastrocnemius MT in male PD patients with sarcopenia compared to their control counterparts. However, it is worth noting that male PD patients with sarcopenia had lower BMI, weight, and MAC. Also, it should be mentioned that the number of male control patients was relatively limited. Akagi et al. (2015) measured a significant increase in stiffness in all regions of the triceps before and after resistance exercise by SWE, and the stiffness varied in different regions. Galvão et al. (2022) found that the elasticity index and strain ratio of spastic muscles were significantly higher in stroke patients than in control subjects. Increased muscle tone in PD patients is one of the main symptoms. The biceps brachii is a flexor muscle of the upper limb, and its contraction causes forearm flexion and supination at the elbow joint. On the other hand, the brachioradialis keeps the forearm in a neutral position and is considered a “regulating muscle.” Consistent with previous studies, we observed that brachioradialis SWE and biceps brachii SWE in a relaxed state were significantly higher in PD patients compared to control subjects (Ding et al., 2021). Furthermore, we found gastrocnemius dorsiflexion SWE was smaller than in control subjects, probably due to the selective nature of dystonia abnormalities in PD patients. The rigidity stems from modifications in the inherent mechanical characteristics of the muscle (Pollock and Davis, 1930). Several studies have demonstrate motor neurons ed that muscles in PD patients display a reduction in either type I or type II fibers when compared to control subjects (Du et al., 2016). The reduction in muscle fibers resulted in a decline in muscle strength, and the gastrocnemius dorsiflexion SWE was diminished. Comparisons between male PD patients with and without sarcopenia showed a decrease in gastrocnemius resting SWE, although it was not statistically significant. However, as shown in Supplementary Table 6, a decrease in gastrocnemius resting SWE was observed when comparing male PD patients with sarcopenia and male control subjects with sarcopenia. We speculate that this decrease during the resting state is likely due to the significant reduction in muscle mass observed in individuals with sarcopenia. Nonetheless, further enlargement of the sample size is necessary to validate this conclusion. The gastrocnemius muscle is an important muscle group in the distal lower limb, playing a crucial role in maintaining knee-ankle joint stability and upright walking. In a study conducted by Gao et al. (2009) on stroke patients with a disease duration of over 1 year, ultrasound findings indicated reduced muscle strength and shorter muscle fiber length, decreased pennation angle, and thinner MT in the affected side compared to normal individuals. Previous studies have demonstrated a strong correlation between forearm MT and overall muscle mass in the limbs or the entire body (Abe et al., 2014, 2018). There is a lack of research on the relationship between the biceps brachii muscle and sarcopenia, as ultrasound assessment for sarcopenia often focuses on measuring forearm muscles in the upper limbs (Ticinesi et al., 2017). Our results confirm that male PD patients with sarcopenia have lower biceps brachii MT while female PD patients with sarcopenia have lower gastrocnemius MT. Several studies have indicated that among various influencing factors, gender has the most significant impact on MT (Abraham et al., 2020). There are differences in the distribution of muscle and adipose tissue between males and females, with males generally having greater MT than females (Janssen et al., 2000). After excluding the influence of muscle strength and physical performance and analyzing only the effect of muscle ultrasound data on muscle mass, as shown in Figure 2, we found that in male PD patients with low ASMI, there was a decrease in both biceps brachii and gastrocnemius MT as well as gastrocnemius fascicle length. Compared with the male PD non-low ASMI group, biceps brachii and gastrocnemius MT as well as gastrocnemius fascicle length were significantly smaller in the PD low ASMI group, which may imply that muscle ultrasound may be a stronger predictor for ASMI.

A national study in Korea in 2014 found that among older adults aged >65 years, the prevalence of sarcopenia was significantly greater in men (46.8%) than in women (7.6%) (Huh et al., 2014). In this study, binary logistic regression analysis revealed that the risk of developing low muscle mass is increased 47 times in male PD patients. Patients who may present with sarcopenia exhibited higher modified H-Y stage and lower ADL scores and higher levels of levodopa equivalent dose (LED) (Lima et al., 2020). Also, a systematic review and meta-analysis of 10 observational studies concluded that sarcopenia is independently associated with depression (Chang et al., 2017). In a recent cross-sectional study including 104 PD patients from a tertiary center in Innsbruck, Austria, sarcopenia was significantly associated with a longer disease duration among other factors (Peball et al., 2019). However, no significant difference was observed between the aforementioned indicators in PD patients with and without sarcopenia in the results of the present study. Nevertheless, PD patients with sarcopenia exhibited a decline in ADL score compared to control patients with sarcopenia, and in male PD patients, brachioradialis SWE increased with the progression of PD. In this study, we observed that in female PD patients, gastrocnemius muscle MT was more correlated with BMI, nutritional status, and body circumference, while in male PD patients, biceps brachii muscle MT was more correlated with the aforementioned indicators. Male PD patients are at a higher risk of developing sarcopenia, and a higher BMI and gastrocnemius MT can prevent PD patients from developing sarcopenia. Overall, these results provide comprehensive insights into the utility of muscular ultrasound assessments in the evaluation of physical performance and muscle strength in PD patients with sarcopenia. The application of muscle ultrasound indices, especially biceps brachii MT, gastrocnemius MT, and SWE, in sarcopenia and the identification of sarcopenia in PD patients holds great significance in the early diagnosis, identification, and intervention to improve patient quality of life and extend lifespan.

This study is not without limitations. The limited number of participants may have resulted in a degree of bias in the findings. We performed multiple two-sample group comparisons based on different criteria, such as gender and the presence of sarcopenia, which can increase the likelihood of Type I errors or false positive results. The absence of dual-energy X-ray absorptiometry (DXA) measurements - the gold standard for diagnosing sarcopenia (Yang and Chan, 2022) - was another limitation. Some of the participants were from outpatients, and it was difficult for us to test both in their OFF and ON states. PD patients with advanced stages were not included in the study as they could not complete the study. To further elucidate the value of muscular ultrasound parameters in PD patients and to establish relevant reference indicators, future studies should aim to expand the sample size considerably. This would address the current limitations and provide a more comprehensive understanding of the relationship between PD and sarcopenia.

## Conclusion

Biceps brachii MT and gastrocnemius MT were found to be useful markers for identifying PD patients with sarcopenia. In PD patients, male patients, low BMI and low gastrocnemius MT were efficient predictors of low muscle mass. Overall, our findings provide important insights into the relationship between PD and sarcopenia. We also illustrated muscle ultrasound measurement of different parts and the techniques which play a crucial role in the assessment of PD with sarcopenia.

## Data availability statement

The raw data supporting the conclusions of this article will be made available by the authors, without undue reservation.

## Ethics statement

The studies involving humans were approved by the Ethics Committee of The Second Affiliated Hospital of Soochow University. The studies were conducted in accordance with the local legislation and institutional requirements. The participants provided their written informed consent to participate in this study.

## Author contributions

MC: Conceptualization, Methodology, Investigation, Data curation, Writing – original draft. XL: Formal analysis, Writing – original draft. QL: Conceptualization, Methodology, Writing – original draft. CD: Investigation, Writing – original draft. PZ: Investigation, Writing – original draft. YZ: Supervision, Writing – review and editing. CM: Conceptualization, Methodology, Writing – review and editing. CL: Conceptualization, Methodology, Supervision, Writing – review and editing.
