# Supplementary material for: The value of ultrasound measurement of muscle thickness at different sites and shear wave elastography in Parkinson’s disease with sarcopenia: a pilot study
Source: Front Neurosci. 2023 Oct 13;17:1254859. doi: 10.3389/fnins.2023.1254859 (PMC10613525; doi:10.3389/fnins.2023.1254859)
Supplement: Supplementary file 1 [file Data_Sheet_1.docx]

**TABLE1 Comparison of PD bilateral muscle ultrasound measurements**

| Muscular ultrasound | Side-of-onset | Non-Side-of-onset | *p* |
| --- | --- | --- | --- |
| Brachioradialis SWE(KPa) ^b^ | 37.09±9.17 | 36.70±8.53 | 0.799 |
| Biceps Brachii SWE(KPa) ^b^ | 67.60±32.69 | 57.47±22.98 | 0.039 |
| Biceps Brachii MT(cm) ^b^ | 2.67±0.45 | 2.65±0.47 | 0.782 |
| Gastrocnemius fascicle length (cm) ^b^ | 4.32±0.65 | 4.42±0.64 | 0.4 |
| Gastrocnemius MT(cm) ^b^ | 1.48±0.21 | 1.51±0.24 | 0.468 |
| Gastrocnemius Pennation Angle (°) ^b^ | 22.86±4.29 | 23.23±4.47 | 0.631 |
| Gastrocnemius Resting SWE(KPa) ^b^ | 17.88±22.38 | 12.75±4.36 | 0.68 |
| Gastrocnemius Dorsiflexion SWE(KPa) ^b^ | 161.18±69.48 | 171.39±71.65 | 0.4 |

Student’s t-test. Values are expressed as mean±standard deviation. MT,Muscle thickness,

SWE,Shear wave elastography.P < 0.05 were considered statistically significant.

**TABLE2 Comparison of HC bilateral muscle ultrasound measurements**

| Muscular ultrasound | Dominant side | Non-dominant side | *p* |
| --- | --- | --- | --- |
| Brachioradialis SWE(KPa) ^b^ | 30.08±6.05 | 35.04±6.85 | 0.001 |
| Biceps Brachii SWE(KPa) ^b^ | 40.65±11.99 | 39.10±12.70 | 0.568 |
| Biceps Brachii MT(cm) ^b^ | 2.67±0.50 | 2.67±0.42 | 0.978 |
| Gastrocnemius fascicle length (cm) ^b^ | 4.46±0.50 | 4.29±0.68 | 0.201 |
| Gastrocnemius MT(cm) ^b^ | 1.56±0.27 | 1.57±0.21 | 0.967 |
| Gastrocnemius Pennation Angle (°) ^b^ | 21.29±3.59 | 22.04±3.51 | 0.336 |
| Gastrocnemius Resting SWE(KPa) ^b^ | 17.10±13.53 | 14.22±5.64 | 0.206 |
| Gastrocnemius Dorsiflexion SWE(KPa) ^b^ | 220.02±46.27 | 231.17±39.93 | 0.241 |

Student’s t-test. Values are expressed as mean±standard deviation. MT,Muscle thickness,

SWE,Shear wave elastography.P < 0.05 were considered statistically significant.

**TABLE3 Clinical characteristics of female PD without sarcopenia and female HC without sarcopenia**

| **Clinical Demographics** | PD-Ns(n=30) | HC-Ns(n=23) | *p* |
| --- | --- | --- | --- |
| Age ^b^ | 62.93±6.90 | 60.65±6.66 | 0.230 |
| SARC-Calf ^b^ | 3.13±4.61 | 1.74±3.88 | 0.248 |
| CC(cm) ^b^ | 34.03±2.61 | 35.39±3.02 | 0.086 |
| WC(cm) ^b^ | 85.85±10.26 | 83.78±7.51 | 0.420 |
| MAC(cm) ^b^ | 27.65±2.29 | 28.80±2.59 | 0.092 |
| Handgrip strength ^b^ | 19.89±5.00 | 24.44±3.78 | **0.001** |
| GS ^b^ | 0.90±0.21 | 1.06±0.15 | **0.003** |
| FTSTS ^b^ | 11.76±3.12 | 9.63±1.94 | **0.006** |
| BMI ^b^ | 24.40±2.40 | 25.56±3.18 | 0.136 |
| Constipation score ^b^ | 6.60±4.51 | 1.14±2.73 | **0.001** |
| Brachioradialis SWE(KPa) ^b^ | 35.21±9.70 | 28.78±5.28 | **0.006** |
| Biceps Brachii SWE(KPa) ^b^ | 65.39±30.73 | 38.39±10.08 | **0.001** |
| Biceps Brachii MT(cm) ^b^ | 2.49±0.42 | 2.49±0.45 | 0.987 |
| Gastrocnemius fascicle length (cm) ^b^ | 4.32±0.73 | 4.43±0.58 | 0.557 |
| Gastrocnemius MT(cm) ^b^ | 1.47±0.16 | 1.64±0.22 | **0.001** |
| Gastrocnemius Pennation Angle (°) ^b^ | 23.21±4.72 | 22.36±3.41 | 0.472 |
| Gastrocnemius Resting SWE(KPa) ^b^ | 18.43±23.13 | 13.60±4.32 | 0.328 |
| Gastrocnemius Dorsiflexion SWE(KPa) ^b^ | 150.19±66.75 | 216.61±50.37 | **0.001** |
| ADL ^c^ | 14.00(14.00,17.00) | 14.00(14.00,14.00) | **0.001** |
| SPPB ^c^ | 11.00(9.00,12.00) | 12.00(11.00,12.00) | **0.001** |

Values are expressed as mean±standard deviation or median (interquartile range). b Student’s t-test. c Mann-Whitney U test. *P<*0.05 were considered statistically significant.Bold values highlight the significant difference.

PD**-**Ns, Parkinson’s Disease without sarcopenia; HC-Ns,Healthy Control without sarcopenia; MT,Muscle thickness,SWE,Shear wave elastography; CC,calf circumference;WC,Waist circumference; MAC,Mid-arm circumference;GS, 6-metre gait speed; FTSTS,Five Times Sit-to-Stand test; BMI,body mass index; ADL, Activity of Daily Living Scale; SPPB,Short Physical Performance Battery.

**TABLE4 Clinical characteristics of male PD without sarcopenia and male HC without sarcopenia**

| **Clinical Demographics** | PD-Ns(n=26) | HC-Ns(n=14) | *p* |
| --- | --- | --- | --- |
| Age (year) ^b^ | 66.38±6.56 | 64.21±3.40 | 0.176 |
| ADL ^b^ | 15.62±3.41 | 14.00±0.00 | **0.023** |
| SARC-Calf ^b^ | 1.08±2.21 | 3.71±5.03 | 0.081 |
| Handgrip strength ^b^ | 28.33±6.87 | 35.87±4.80 | **0.001** |
| SPPB ^b^ | 10.08±2.10 | 11.71±0.83 | **0.001** |
| Constipation score ^b^ | 6.27±4.84 | 0.27±0.65 | **0.001** |
| Brachioradialis SWE(KPa) ^b^ | 38.62±8.07 | 32.40±6.93 | **0.019** |
| Biceps Brachii SWE(KPa) ^b^ | 70.56±35.48 | 42.56±13.03 | **0.001** |
| Biceps Brachii MT(cm) ^b^ | 2.99±0.32 | 3.09±0.34 | 0.369 |
| Gastrocnemius fascicle length (cm) ^b^ | 4.45±0.56 | 4.59±0.35 | 0.373 |
| Gastrocnemius MT(cm) ^b^ | 1.57±0.22 | 1.56±0.25 | 0.950 |
| Gastrocnemius Pennation Angle (°) ^b^ | 23.01±3.51 | 20.69±3.10 | **0.045** |
| Gastrocnemius Resting SWE(KPa) ^b^ | 18.82±25.99 | 19.47±20.42 | 0.936 |
| Gastrocnemius Dorsiflexion SWE(KPa) ^b^ | 166.50±75.20 | 219.57±46.85 | **0.009** |

Values are expressed as mean±standard deviation. b Student’s t-test. *P* < 0.05 were considered statistically significant.Bold values highlight the significant difference.

PD**-**Ns, Parkinson’s Disease without sarcopenia; HC-Ns,Healthy Control without sarcopenia; MT,Muscle thickness;SWE,Shear wave elastography. ADL, Activity of Daily Living Scale; SPPB,Short Physical Performance Battery.

**TABLE5 Clinical characteristics of PD with and without sarcopenia**

| **Clinical Demographics** | PD**-**s**(n=12)** | PD-Ns**(n=56)** | *p* |
| --- | --- | --- | --- |
| SARC-Calf ^b^ | 8.92±5.44 | 2.18±3.81 | **0.001** |
| CC(cm) ^b^ | 31.88±1.81 | 35.39±2.76 | **0.001** |
| WC(cm) ^b^ | 78.67±9.30 | 88.11±9.56 | **0.003** |
| MAC(cm) ^b^ | 25.71±1.36 | 28.39±2.32 | **0.001** |
| BMI ^b^ | 20.17±1.43 | 24.66±2.84 | **0.001** |
| UPDRS I ^b^ | 4.58±2.47 | 3.05±1.92 | **0.020** |
| ASMI ^b^ | 6.10±0.76 | 7.05±0.89 | **0.001** |
| Weight (kg) ^b^ | 53.33±5.48 | 64.89±8.67 | **0.001** |
| WHR ^b^ | 0.83±0.03 | 0.88±0.04 | **0.001** |
| Gastrocnemius MT(cm) ^b^ | 1.32±0.16 | 1.51±0.20 | **0.002** |
| GS ^c^ | 0.73(0.64,0.90) | 0.92(0.82,1.03) | **0.008** |
| FTSTS ^c^ | 13.07(10.19,25.98) | 11.20(9.37,13.88) | 0.053 |
| SPPB ^c^ | 9.00(8.00,10.50) | 11.00(9.00,12.00) | 0.062 |
| MNA ^c^ | 11.00(9.00,11.75) | 13.00(12.00,13.00) | **0.001** |

Values are expressed as mean±standard deviation or median (interquartile range). b Student’s t-test. c Mann-Whitney U test.P < 0.05 were considered statistically significant.Bold values highlight the significant difference.

PD**-**s, Parkinson’s Disease with sarcopenia; PD**-**Ns, Parkinson’s Disease without sarcopenia; CC,calf circumference; WC,Waist circumference;MAC,Mid-arm circumference; BMI,body mass index; UPDRS I, Unified Parkinson’s Disease Rating Scale part I; ASMI,appendicular skeletal muscle index; WHR,Waist-to-Hip Ratio; GS, 6-metre gait speed; FTSTS,Five Times Sit-to-Stand test; SPPB,Short Physical Performance Battery; MNA,Mini-Nutritional Assessment;MT,Muscle thickness.

**TABLE6 Clinical characteristics of male PD with sarcopenia and male HC with sarcopenia**

| **Clinical Demographics** | PD-s(n=7) | HC-s(n=3) | *p* |
| --- | --- | --- | --- |
| Age (year) ^b^ | 69.29±7.30 | 74.67±8.62 | 0.338 |
| MAC(cm) ^b^ | 25.93±1.17 | 24.17±0.29 | **0.037** |
| ASMI ^b^ | 6.67±0.21 | 6.32±0.23 | **0.046** |
| Weight (kg) ^b^ | 56.61±4.65 | 49.53±1.54 | **0.037** |
| Brachioradialis SWE(KPa) ^b^ | 37.01±8.53 | 31.12±3.49 | 0.293 |
| Biceps Brachii SWE(KPa) ^b^ | 66.21±25.58 | 47.75±19.87 | 0.302 |
| Biceps Brachii MT(cm) ^b^ | 2.62±0.26 | 2.51±0.29 | 0.582 |
| Gastrocnemius fascicle length (cm) ^b^ | 4.11±0.72 | 4.13±0.45 | 0.963 |
| Gastrocnemius MT(cm) ^b^ | 1.40±0.13 | 1.09±0.25 | **0.030** |
| Gastrocnemius Pennation Angle (°) ^b^ | 22.31±5.65 | 17.00±4.58 | 0.192 |
| Gastrocnemius Resting SWE(KPa) ^b^ | 12.86±4.39 | 24.64±10.22 | **0.028** |
| Gastrocnemius Dorsiflexion SWE(KPa) ^b^ | 166.60±68.67 | 229.44±21.29 | 0.17 |

Values are expressed as mean±standard deviation. b Student’s t-test.*P*<0.05 were considered statistically significant.Bold values highlight the significant difference.

PD**-**s, Parkinson’s Disease with sarcopenia; HC-s,Healthy Control with sarcopenia; MT,Muscle thickness,SWE,Shear wave elastography. MAC,Mid-arm circumference; ASMI,appendicular skeletal muscle index.

**
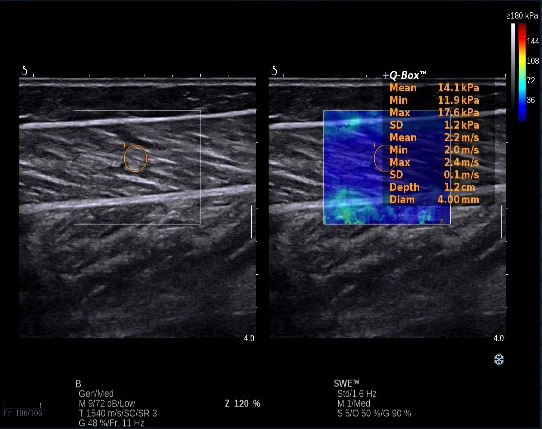

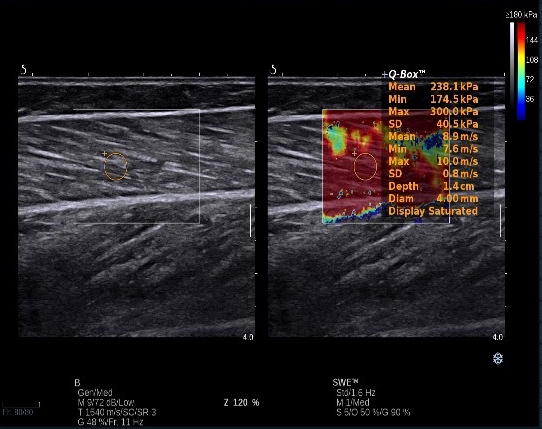
**

**A B**

**FIGURE1 Shear wave elastography sample results of Gastrocnemius**

A.Shear wave elastography sample results of Gastrocnemius Resting SWE, a circle denotes the region of interest, the mean SWE was 14.1KPa. B.Shear wave elastography sample results of Gastrocnemius Dorsiflexion SWE, the mean SWE was 238.1KPa.
